# Effect of *Vietnamese coriander* Powder on Growth, Body Composition, Hematology, and Immune-Related Gene Expression in Nile Tilapia

**DOI:** 10.1155/vmi/1253764

**Published:** 2025-02-26

**Authors:** Apisara Honghirun, Rungrawee Thongdon-a, Niran Aeksiri, Kumrop Ratanasut, Wilasinee Inyawilert, Gen Kaneko, Anurak Khieokhajonkhet

**Affiliations:** ^1^Department of Fisheries, Rajamangala University of Technology Lanna Phitsanulok, Phitsanulok, Thailand; ^2^Center for Agriculture Biotechnology, Naresuan University, Phitsanulok, Thailand; ^3^College of Natural and Applied Science, University of Houston-Victoria, Victoria, Texas, USA

**Keywords:** growth performance, herb, immunity, Nile tilapia, *Persicaria odorata*

## Abstract

Dietary supplementation of plant herbs into aquafeed is recommended for intensive and sustainable aquaculture practices. This study has investigated the effect of *Persicaria odorata* (PO) leaf powder on growth, feed utilization, whole-body composition, hematology, and immune-related gene expression in Nile tilapia (*Oreochromis niloticus*). Fish (∼7.77 ± 0.01 g/fish) were randomly allocated into four treatment groups, each with three replicates. They were fed with dietary supplementation of PO at concentrations of 0, 15, 30, and 50 g/kg (termed PO0–PO50) for 10 weeks. Results indicated that the final body weight, weight gain, and specific growth rate were significantly higher at all PO supplementation levels compared to the control, with the highest value in the PO30 group. Similarly, the protein efficiency ratio and protein productive value in all PO groups were significantly higher than those of the control group. Red blood cells, white blood cells, and hemoglobin levels showed quadratic increases in the PO groups. In addition, total protein, globulin, and high-density lipoprotein cholesterol were linearly and quadratically increased with increasing PO levels, but alanine aminotransferase activity was linearly and quadratically decreased. Furthermore, dietary PO supplementation linearly decreased triglyceride and lipoprotein cholesterol levels, with the lowest levels found in the PO15 group. The expression of genes related to immunity showed that dietary supplementation of PO significantly increased the expression of proinflammatory factors (IL-1β and TNF-α), anti-inflammatory (IL-10 and TGF-β), and HSP70. In addition, glucose and cortisol levels decreased in all PO-supplemented groups, with the lowest levels found in the PO50 and PO30 groups, respectively. These findings showed that a dietary intervention with PO could improve growth, feed utilization, hematological parameters, upregulating genes related to immunity, and decreasing stress markers in Nile tilapia. Consequently, the utilization of PO at a dosage of 30 g/kg could contribute to the sustainable development of the aquaculture sector.

## 1. Introduction

Aquaculture stands as a lucrative and swiftly expanding industry. It plays a vital role in ensuring human food security by producing high-quality aquatic products [1, 2]. Nevertheless, the recent expansion of intensive aquaculture has been linked to the risk of various diseases, which pose significant financial threats to fish farms and the aquaculture industry as a whole [3]. Currently, a variety of preventive and treatment strategies have been proposed to combat fish diseases such as the utilization of chemical drugs, vaccines, and antibiotics [2, 4]. However, these materials are often made more complex by the use of different trade names and the varying names of active compounds [2, 5]. For example, a recent study revealed the use of an average of 28.8 chemical and biological substances per farm, with a total of 263 different products recorded in Bangladesh [5]. A study in India recorded 364 aquamedicines, drugs, and chemicals available to farmers for combating diseases in 2017 [6]. However, there are several limitations to the use of vaccines and antibiotics in aquaculture [4, 7]. Vaccine efficiency is varied by environmental factors, and there is a risk of pathogen mutation. Injection and implantation can cause handling stress and require intensive labor, particularly in large-scale fish farming operations [4, 8]. In addition, health concerns regarding the drug residues in fish and the environment, along with the resultant antimicrobial resistance, pose significant limitations for the use of antibiotics and chemicals in aquaculture [2]. Therefore, compounds derived from medicinal plants are more easily broken down compared to synthetic drugs, aligning with the key goal of sustainable food production to reduce the ecological burden on natural resources [1, 9].

An immunostimulant is a natural or chemical substance that enhances the immune system through either specific (vaccines or antigens) or nonspecific (irrespective of antigenic specificity) routes [10]. In aquaculture, nonspecific immunostimulants are widely used, likely due to the limited understanding of the fish immune response and the ease of application and seems to be the most practical [10]. Numerous studies have examined the use of dietary immunostimulants to prevent and treat infectious diseases in aquatic species as effective alternatives to vaccines and antibiotics [4, 11, 12]. Herbs are the representative functional feed ingredients that have been utilized as immunostimulants in traditional medicine for several decades [11, 13]. They contain a wide range of nutritional components and active substances such as polysaccharides, alkaloids, or flavonoids, minerals, and vitamins. These immunostimulants can boost nonspecific immune response in fish, which strengthen immunological responses and improve disease resistance [10]. Currently, herbs are used in commercial aquaculture for a variety of purposes, including nutrients, growth-promoting agents, and antibacterial compounds. In addition, they can enhance the efficacy of vaccinations, making the immunization process more effective [11, 14]. *Persicaria odorata* (PO), commonly known as Vietnamese coriander, is a widely used herb from the Polygonaceae family, which is frequently utilized in Asian cuisine, particularly in Malaysia, Thailand, Singapore, and Vietnam [15]. PO possesses a distinctive pungent aroma that imparts a unique flavor to dishes when used [16]. In traditional medicine, the leaf of PO has been employed to treat ailments such as inflammation and diarrhea, attributing their efficacy to the diverse biological properties resulting from the presence of volatile compounds [15]. The PO is rich in several active compounds with pharmacological properties [17–20]. The most prevalent active compounds found in PO include polyphenols (32.17–58.56 μg/mg), flavonoids (70.65 μg/mg), quercetin (7.20 g/100 g), β-caryophyllene (36.5 g/100 g), decanol (4.9 g/100 g), dodecanal (11.4 g/100 g), and caryophyllene oxide (8.2 g/100 g) [21–23]. Previous studies have also indicated that essential oil derived from PO effectively inhibits various bacteria, including *Salmonella choleraesuis*, *Enterococcus faecalis*, *Enterococcus faecium*, *Staphylococcus epidermidis*, and *Staphylococcus aureus* [18]. Research on fresh leaf, essential oil, and its extracts confirmed its antibacterial properties, and extracts from PO displayed significant antifungal activity [24] as well as antimicrobial, anti-inflammatory, antitumor, anticancer, antiviral, and antioxidant properties [17, 18]. PO also showed astringent qualities, 5α-reductase inhibition, and antioxidative ability against hydrogen peroxide and ABTS radicals [25].

Nile tilapia (*Oreochromis niloticus*) is globally recognized as a crucial aquaculture species [26] owing to its rapid growth rate, relatively high tolerance to biotic and abiotic stressors, superior meat quality, rising consumer preference, and high market value [27]. However, the tilapia farming industry faces significant challenges due to bacterial, viral, and parasitic diseases, which severely impact its production. Among these pathogens, the pathogenic bacteria, *Streptococcus agalactiae*, is a significant bacterial threat to Nile tilapia, emerging as the primary bacterial pathogen affecting this species [28]. *S. agalactiae* produces the cAMP factor, a pore-forming and cytolytic toxin. The functions of these proteins might contribute to the similar pathogenesis of group B *Streptococcus* (GBS) in fish [29], enhancing the hemolytic activities of sphingomyelinase (β-toxin) [30]. These factors cause substantial mortality rates, resulting in significant economic losses of Nile tilapia on a global scale [31]. Achieving sustainable tilapia aquaculture requires the implementation of strategies aimed at enhancing fish health. Therefore, this study marks the first attempt to assess how dietary supplementation with PO affects growth performance, feed utilization, body composition, and hematology in Nile tilapia. This study also examined the potential advantages of PO in enhancing fish immunity through the evaluation of genes associated with the immune system. To the best of our knowledge, PO has demonstrated the ability to improve growth, feed utilization, hematological parameters, histological characteristics, and meat quality in terrestrial animals such as broiler chickens [32–34]. Nevertheless, no studies have explored its effects on teleost fish species. The present study has shown that the application of this medicinal herb in fish diets could prove beneficial in disease control and represents a step toward more sustainable aquaculture.

## 2. Materials and Methods

### 2.1. Preparation of PO

Fresh PO was purchased at a local wholesale market in Phitsanulok Province, Thailand. The herbal samples were washed with tap water twice and shade-dried for 2 h. Cleaned PO was sliced approximately 2-3 cm long and subjected to a hot-air oven at 50°C overnight. Thereafter, the PO was finely ground using a kitchen blender. Finally, the PO powder was sieved using a mesh (600 μm) and stored in plastic zip bags at −20°C until used for feed formulation.

### 2.2. Diet Preparation

In this study, four isonitrogenous (∼31%) and isolipidic (∼7%) diets were prepared. The basal diet contained 135 g/kg of fish meal, 400 g/kg of soybean meal, and 22 g/kg of soybean oil as the major feed component. Soybean oil was used in the present study because it has no effect on growth and serves as a good source of polyunsaturated fatty acids for Nile tilapia [35]. In addition, lecithin was also supplied at 4 g/kg to all diet treatments because it provides a vital source of bioavailable phospholipids for aquatic animals [36], supporting energy production through nutrient metabolism and serving as a key component of biological membranes in Nile tilapia [37, 38]. The PO powder was supplemented to the basal diet at 15, 30, and 50 g/kg corresponding to PO15, PO30, and PO50, respectively (Table 1). In brief, all powder ingredients of the experimental diets were weighed, mixed, and homogenized for 15 min using a CKI family kitchen homogenizer (Nonthaburi, Thailand). After homogenizing with soybean oil and lecithin for 5 min, distilled water was added in a 35% proportion to form a soft feeding dough. A meat mincer was used to form the feeding pellets of 3 mm diameter, which were cut to approximately 2-3 mm long. The prepared experimental diets were then kept in a plastic bag and stored at −20°C until used for proximate analysis and feeding.

### 2.3. Experimental Design

The fingerling Nile tilapia (*Oreochromis niloticus*) were procured from a local commercial freshwater fish hatchery (DokDin hatchery), Phitsanulok, Thailand. Fish were manually fed until apparent satiation with a commercial diet for Nile tilapia containing 30% crude protein and 4% crude lipid and were adopted in a laboratory condition for 2 weeks. Thereafter, apparently healthy fish (240 fish, 7.77 ± 0.01 g/fish) were randomly allocated into 4 triplicate groups, each of which was kept in a 250-L tank filled with 200 L dechlorinated water (20 fish per tank, total of 12 tanks). Nile tilapia were fed until apparent satiation twice daily (8.30 and 17.30) during the experimental period (10 weeks) under the natural photoperiod (∼12-h light:∼12-h dark). During the feeding trial, water was siphoned off to remove feces and debris every day, and 30% of water was exchanged with dechlorinated water. Water parameters were determined twice a week; temperature ranged from 26.80°C to 28.10°C, dissolved oxygen 5.4–6.8 mg/L, and pH 7.1–7.9.

### 2.4. Sampling Strategies

#### 2.4.1. Growth Performance and Organosomatic Parameters

At the end of the 10-week period, Nile tilapia were deprived for 24 h. Subsequently, they were rendered using clove oil solution (30 ppm) composed of a mixture of 1 part clove oil to 9 parts ethanol. Each replicate (three replicates per treatment) was counted and weighed in bulk to determine weight gain (WG, g/fish), specific growth rate (SGR, %/day), feed conversion ratio (FCR), and survival as described in our previous report [41]. Three fish from each tank (*n* = 9) were randomly collected and individually measured for the total length and weight to determine the condition factor (*K* value, g/cm^3^). Fish was euthanized using an overdose of the clove oil mixture solution and excised to obtain visceral and liver organs to determine viscerosomatic index (VSI, %) and hepatosomatic index (HSI, %) as described in our previous report [41].

#### 2.4.2. Chemical Analyses

Moisture, crude lipid, crude protein, and ash of experimental diets and the whole body of fish were determined by following our previous study [41]. The moisture content of the samples was determined by drying them in a hot-air oven at a temperature of 105°C until a constant weight was achieved. The crude protein content was determined by following the Kjeldahl method using semiautomatic Kjeldahl, Gerhardt Vapodest, 45 s (Germany). The crude lipid content of the samples was determined by extracting lipids using a classical Soxhlet apparatus with chloroform solvent (Gerhardt, Germany), and ash content was analyzed by incinerating in a muffle furnace at 550°C for 8 h. The crude fiber was determined by digestion with acid (H_2_SO_4_) and base (NaOH), followed by incineration at 525°C for 3 h using a muffle furnace.

#### 2.4.3. Hematological and Biochemical Analyses

The blood samples were withdrawn from the caudal vein of three fish per replicate (pooled by tank, *n* = 3 per treatment) using a 25-G syringe with 10% ethylenediaminetetraacetate (EDTA) for analyzing hematological parameters. Red blood cells (RBCs, cell/μl) and white blood cells (WBCs, cell/μl) were counted using a Neubauer hemocytometer by following our previous study [41]. The total hemoglobin (Hb, g/dL) concentration was determined using a Drabkin's colorimetric kit by measuring the absorbance of the samples at a wavelength of 540 nm. The hematocrit (Hct, %) values were determined by subjecting the blood samples to centrifugation in a microhematocrit centrifuge (DLAB, Beijing, China) at a speed of 12,000 rpm for 5 min. Blood glucose content (nmol/L) was determined using a blood glucose meter, Accu-Check Active Glucometer, Roche (Mannheim, Germany).

Another set of fish blood samples (three fish per tank were pooled, *n* = 3 per treatment) were collected without anticoagulant and placed on ice for 1 h to allow to clot. To collect blood serum, the blood samples were subjected to centrifugation at 4°C for 15 min with a speed of 2000 x g. The upper layer was collected and stored at −80°C until used for serum biochemical analysis. The biuret method [42] and bromocresol green method [43] were employed to measure the levels of total serum protein (g/dL, at a wavelength of 550 nm) and albumin (g/dL, at a wavelength of 626 nm), respectively. Globulin was calculated by using the following equation: total globulin (g/dL) = total serum protein − total albumin. The activities of aspartate aminotransferase (AST, U/L, at a wavelength of 546 nm), alanine transaminase (ALT, U/L, at a wavelength of 550 nm), and alkaline phosphatase (ALP, U/L) and the concentration of triglycerides (mg/dL, at a wavelength of 510 nm), cholesterol (mg/dL, at a wavelength of 410 nm), high-density lipoprotein cholesterol (HDL-c, mg/dL, at a wavelength of 600 nm), and low-density lipoprotein cholesterol (LDL-c, mg/dL, at a wavelength of 600 nm) in serum were analyzed by using colorimetric methods [44] with a blood biochemical analyzer (Roche Diagnostics, Switzerland). A commercial enzyme-linked immunosorbent assay (ELISA) kit (Wuhan, China) was used to analyze serum cortisol (ng/mL), with the final measurement taken at an absorbance of 450 nm.

### 2.5. Total RNA Extraction and Reverse Transcription

Two Nile tilapia were randomly collected from each tank (*n* = 6 per treatment) and euthanized with an overdose (50 ppm) of the clove oil mixture solution. Fresh liver tissues of approximately 2 g were collected and immediately minced with a pestle and mortar in liquid nitrogen until fine powder. Samples were homogenized with Qiazol Lysis Reagent (Qiagen, Maryland, USA) and subjected to purification using RNeasy Mini Kit, Qiagen (Hilden, Germany), by following the instruction guidelines. To remove genomic DNA, total RNAs were incubated with DNase I (Thermo Fisher Scientific, Waltham, USA) by following the manufacturer's instructions. Total RNAs were determined for the quantity and integrity by measuring the 260/280-nm absorbance and by 2% agarose gel electrophoresis, respectively. Purified RNAs were reverse-transcribed using the RevertAid First-Strand Synthesis System, Thermo Scientific (Waltham, MA, USA) by following the manufacturing's protocol.

### 2.6. Quantitative Real-Time PCR (qRT–PCR) Analysis of Genes to Related Immunity

The qRT-PCR was performed to determine the expression of genes related to immunity including interleukin-1β (IL-1β), IL-10, tumor necrosis factor-alpha (TNF-α), transforming growth factor-alpha (TGF-α), and heat shock protein-70 (HSP70). IL-1β, IL-10, and TNF-α were designated according to the published sequences [45–47], respectively. The HSP70 gene has been annotated as HSP70cB1, following the latest nomenclature [48]. The TGF-β and β-actin were designed using Primer Premier 3.0, based on the nucleotide sequences of those genes in Nile tilapia, with accession number provided in Table 2. qPCR was performed with 20 μL total volume of mixture solution containing 1 μL of 100X diluted template, 0.5 μL of 10 μM of forward and reverse primers, 10 μL of Maxima SYBR Green/ROX qPCR Master Mix (2X), Thermo Fisher Scientific, (Pittsburg, PA, USA), and 8 μL of double distilled water. Each sample was analyzed by qPCR consisting of 95°C for 3 min of denaturation followed by 40 cycles of 95°C for 10 s and 60°C for 30 s. qPCR was performed in triplicate using PCRmax ECO48 Real-time qPCR system (Staffordshire, UK). The β-actin gene was used as an internal control, and the relative mRNA expression levels were determined using the 2^−ΔΔCT^ method.

### 2.7. Statistical Analysis

The experimental data were reported as mean ± standard deviation, and statistical analysis was performed using SPSS 17.0 software (Chicago, IL, USA) with the accepted significance level set up at *p* < 0.05. The results were compared using a one-way ANOVA. Orthogonal polynomial contrasts were calculated to detect the linear and quadratic effects of different levels of PO supplementation. In addition, Dunnett's test was applied to determine differences between the control group and the PO supplementation groups.

## 3. Results

### 3.1. Growth Performance, Survival, and Organosomatic Indexes

**Figure 1:**
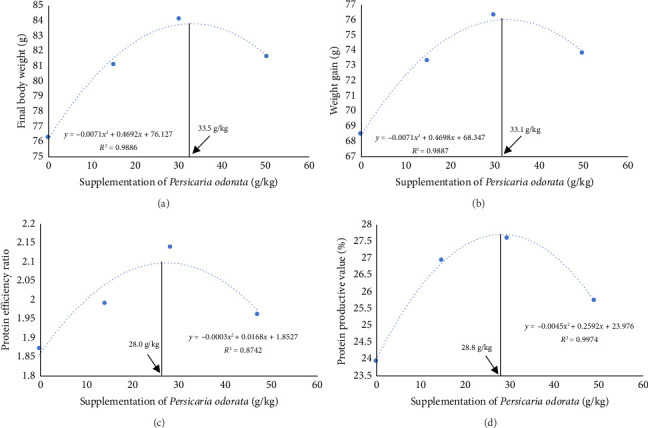
Second-order polynomial relationship of final body weight (a), weight gain (b), protein efficiency ratio (c), and protein productive value (d) to dietary supplementation of *Persicaria odorata* (PO) of Nile tilapia.

The growth parameters, feed utilization, and organosomatic indexes of Nile tilapia fed diet supplemented with PO at different concentrations are shown in Table 3. During the feeding trial for 10 weeks, all growth and feed utilization parameters linearly or quadratically increased with increasing PO levels (ANOVA, *p* < 0.05). Final body weight (76.28–84.16 g/fish) increased to about 10-fold compared with IBW (∼7.77 g/fish) in all groups. Dunnett's test revealed a noteworthy elevation in FBW, WG, and SGR from the PO0 group across all levels of PO supplementation with the highest values observed in the PO30 group (Dunnett's test *p* < 0.05). For FCR, only the PO50 group had a significant increase compared to the control group (Dunnett's test, *p* < 0.05), although the linear and quadratic effects were significant (ANOVA, *p*=0.009; *p*=0.028). Protein utilization (PER and PPV) was linearly and quadratically increased with increasing PO levels (ANOVA, *p* < 0.001). All PO-supplemented groups showed significant increases in PER and PPV compared to the PO0 group, and the highest PER and PPV values were found in the PO30 group (Dunnett's test; *p* < 0.05). Organosomatic indexes including K value, HIS, and VSI showed no linear or quadratic effects of PO levels (ANOVA, *p* > 0.05). The results of the second-order polynomial regression analysis indicate that the highest FBW, WG, PER, and PPV can be attained at PO supplementation levels of 33.5%, 33.1%, 28.0%, and 28.8%, respectively (Figures 1(a), 1(b), 1(c), 1(d)).

### 3.2. Whole-Body Proximate and Chemical Composition

Dietary PO supplementation resulted in a significant quadratic increase in crude protein content in Nile tilapia (ANOVA, *p*=0.017; Table 4). Fish fed PO15 and PO30 diets showed significantly higher crude protein content compared to the control group (Dunnett's test, *p* < 0.05), with the highest level observed in the PO30 group. The whole-body crude fat content exhibited linear and quadratic responses to the dietary PO levels (ANOVA, *p* < 0.001; *p* < 0.001), with the PO30 group showing the highest value, which was significantly greater than that of the control group (Dunnett's test, *p* < 0.05). However, no significant alterations were observed in the moisture and ash contents among the various dietary treatments (ANOVA, *p* > 0.05; Table 4).

### 3.3. Hematological Profile

The hematological parameters of the fish fed different levels of PO supplementation are depicted in Table 5. No significant difference in Hct was observed among different levels of PO supplementation groups (ANOVA, *p*=0.073; *p*=0.091). However, RBC, WBC, and Hb were quadratically increased by PO supplementation (ANOVA, *p* < 0.05). RBC and Hb were found to be higher in the PO50 and PO30 groups (Dunnett's test, *p* < 0.05, Table 5), and WBC was significantly increased in the PO15 and PO50 groups compared to the control group (Dunnett's test, *p* < 0.05).

### 3.4. Blood Biochemical Parameters

At 10 weeks, dietary supplementation of PO resulted in linear and quadratic increase in total protein (ANOVA, *p*=0.001; *p*=0.035, Table 6). The PO50 group exhibited higher values than those of the other groups and was significantly greater compared to the control group (Dunnett's test, *p* < 0.05). Globulin levels exhibited the same tendency as total protein (ANOVA, *p* < 0.001; *p*=0.005), while both PO30 and PO50 significantly increased the value compared to the control diet (Dunnett's test, *p* < 0.05). The levels of albumin and A:G ratio showed no linear or quadratic response to PO supplementation levels (ANOVA and Dunnett's test, *p* > 0.05). Fish fed with PO-enriched diets displayed significant linear and quadratic decreases in ALT levels (ANOVA, *p*=0.018; *p*=0.007) but not in AST and ALP levels (ANOVA, *p* > 0.05). The PO supplementation levels in diet quadratically decreased triglycerides (ANOVA, *p*=0.020) and LDL-c (ANOVA, *p*=0.003) and linearly and quadratically increased HDL-c levels (ANOVA, *p*=0.002; *p*=0.002). According to Dunnett's test, the PO30 and PO50 groups showed significantly higher HDL-c levels than the control group (Dunnett's test, *p* < 0.05; Table 6).

### 3.5. Relative mRNAs Expression

**Figure 2:**
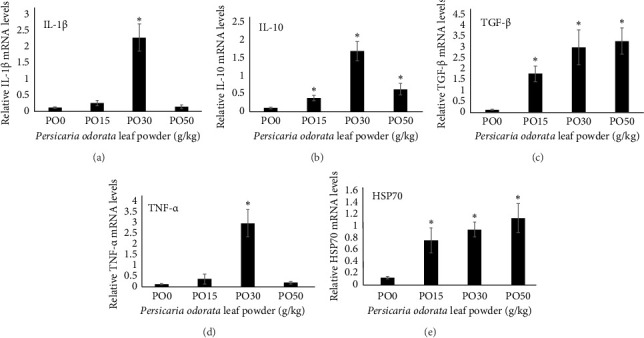
Effect of dietary *Persicaria odorata* (PO) supplementation on gene expression; IL-1β, IL-10, TGF-β, TNF-α, and HSP70 of Nile tilapia for 10 weeks: Diet 1 (control, 0 g/kg PO), Diet 2 (15 g/kg PO), Diet 3 (30 g/kg PO), and Diet 4 (50 g/kg PO). The data in the study are expressed as the mean values for six individuals per treatment (*n* = 6). In Dunnett's test, significant differences are indicated by an asterisk (*p* < 0.05).

Dietary supplementation of PO significantly increased the expression of genes related to immunity (IL-1β, TNF-α, TGF-β, and IL-10) and stress (HSP70) in the liver of Nile tilapia (Figures 2(a), 2(b), 2(c), 2(d), and 2(e)). The expression of IL-1β, IL-10, and TNF-α mRNA in the tilapia hepatic tissue was significantly higher in the PO30 group (Figures 2(a), 2(b), and 2(d)) compared to the control group (*p* < 0.05, Dunnett's test). Similarly, PO significantly increased the expression of TGF-β and HSP70 (Figures 2(c) and 2(e)) at all supplementation levels compared to the control group (Dunnett's test, *p* < 0.05). The highest expression of levels of TGF-β and HSP70 genes were observed in the PO30 and PO50, respectively (Figures 2(c) and 2(e)).

### 3.6. Blood Glucose and Cortisol Levels

The supplementation of PO in diets led to a linear reduction in glucose levels (ANOVA, *p*=0.010; *p*=0.071; Table 7), with the lower level in the PO50 group when compared with a control group (Dunnett's test, *p* < 0.05). In addition, dietary supplementation with PO linearly and quadratically decreased cortisol levels (ANOVA, *p* < 0.001; *p*=0.016). Dunnett's test revealed a noteworthy reduction in cortisol at all levels of PO supplementation (Dunnett's test; *p* < 0.05).

## 4. Discussion

The future of the aquaculture industry lies in the utilization of functional feeds [12, 49]. Within the realm of aquaculture, various medicinal plants have been incorporated into feed as functional additives, employing various forms such as the entire plant, specific plant components such as leaf, roots, or seeds, or even isolated compounds extracted from these plants [12]. These approaches have generated a great deal of attention to the well-being effects on fish including growth improvement, antioxidant, lipid-lowering, immunostimulant, and disease resistance [12, 50, 51]. Many studies reported that plant-based supplements or their extracts in diets may modulate immune responses in a time- and dose-dependent manner [52], with typically becoming evident after 4–12 weeks of supplementation [52, 53]. These medicinal plants and their derivatives are emerging as promising sustainable solutions for disease prevention and control in aquaculture. A present study aimed to investigate the effects of dietary supplementation of PO on growth, feed and nutrient utilization, blood biochemical hematological indices, expression of genes related to immunity, and stress markers in Nile tilapia for 10 weeks.

In the present study, dietary supplementation of PO at 15, 30, and 50 g/kg linearly and quadratically increased the growth performance (FBW, WG, and SGR) within 10 weeks. In addition, all PO-supplemented groups exhibited significantly higher growth performance compared to the control group, with the highest values observed in the PO30 group (*p* < 0.05). These results might be attributed to the bioactive compounds in PO [22, 54]. It has been documented that PO is rich in quercetin and eugenol [54]. These compounds process the ability to promote the biological development in Nile tilapia [55–58]. Although there have been no investigations about the effects of PO supplementation on Nile tilapia, dietary supplementation of 2 and 8 g/kg PO powder and 200–600 mg/kg PO extracts significantly increased final body weight and average daily gain in terrestrial animals such as broiler chickens [33, 34, 59]. The results obtained in this study align with the finding from other vegetable leaf, such as coriander and basil, which have shown that inclusion levels of 10–30 g/kg can achieve optimal growth performance in various fish species [60–62]. However, supplementation higher than 50 g/kg of basil leaf adversely negatively affected growth and other physiological traits in catfish (*Clarias gariepinus*) [63]. Dietary inclusion of basil leaf at 0–150 g/kg did not impair growth performance in common carp (*Cyprinus carpio*) and catfish (*Clarias gariepinus*) [64, 65], though a 150 g/kg group reduced growth, feed utilization, and increased mortality in catfish [65]. These discrepancies may be attributed to factors such as fish species, fish age, and the presence of antinutritional compounds.

In the present study, dietary supplementation of PO linearly and quadratically increased FCR (*p* < 0.05), although a significant increase was observed only in the PO50 group. Many studies have revealed that herbal plants improve animal growth and feed utilization, but it is essential to note that excessive supplementation of herbal plants may introduce antinutritional factors into feeds, potentially resulting in growth retardation and diminished feed utilization [66–68]. According to a recent study [22], PO contains antinutritional factors such as tannins, alkaloids, and saponins. Protein utilization, such as PER and PPV, are indicators of protein quantity, quality, and amino acid balance in fish diets. These parameters are used to evaluate protein utilization and turnover, as they reflect the relationship between dietary protein intake and its conversion into fish growth and protein gain [69, 70]. In the present study, all dietary supplementation of PO linearly and quadratically increased protein utilization (PER and PPV), and the PO30 group showed superior PER and PPV compared to the other groups (*p* < 0.05), demonstrating that PO could potentially spare protein for growth in Nile tilapia. Organosomatic indexes, condition factor, HSI, and VSI, have been used as somatic biomarkers for evaluating the nutritional status, health status, and physiological conditions in fish [71]. In the present study, dietary supplementation with varying levels of PO exhibited no significant influence on these factors. A similar finding has been reported, showing that dietary supplementation with various herbal plants, such as mulberry leaf, *Moringa oleifera* leaf, and a combination of plant leaf meal, did not affect the weight of the liver and abdominal fat [72–74]. These results indicate that there is no significant difference in HSI, VSI, and K values among the groups which may be attributed to nonaccumulation of fat in the visceral and liver organs, or it may indicate that the fat metabolism and overall physiology of Nile tilapia on the PO group remains unaffected.

The nutritional composition and quality of the diet, feeding levels, feeding regime, and various other factors can all influence the body proximate composition of fish [75]. In the present study, whole-body crude protein and crude fat content quadratically increased by PO supplementation (Table 4). These changes may arise from the bioactive compounds, such as flavonoids, in the PO. A previous study demonstrated that flavonoids in diet effectively controlled the uptake, transportation, and metabolism of lipids in tilapia [76] associated with the expression change of genes related to fatty acid synthesis and metabolism, NF-κβ, c-Rel, and MAPK8. These bioactive constituents have the capacity to stimulate metabolism, enhancing the mobilization and breakdown of lipids as well as the efficiency of protein synthesis. Furthermore, Nile tilapia fed with an *Elephantopus scaber* extract, known for its high flavonoid content, exhibited elevated levels of crude lipid and crude protein in their carcasses [77].

Scientific methods to assess fish welfare in aquaculture are evolving, focusing on how fish respond to environmental or human-induced stressors. Various factors, such as nutritional status and social stress, can disrupt homeostasis, prompting fish to initiate responses to restore balance [78]. The initial stress response involves catecholamine release and activation of the hypothalamic–pituitary–interrenal axis, leading to corticosteroid release. This triggers increased cardiorespiratory activity, altered tissue oxygen demands, blood changes, and energy mobilization and affects the physiological well-being of cultured fish [26, 79]. In the present study, supplementing the diet with PO had quadratic impacts on RBC, WBC, and Hb levels. RBC and Hct levels showed a positive trend with increasing PO levels, although no significant difference was observed in Hct. Generally, increased RBC and Hct levels enhance the blood's oxygen-carrying capacity, improving oxygen affinity and transport in the circulatory system [80]. Paschko et al. [81] reported that PO serves as a source of essential minerals, including phosphorus, potassium, magnesium, and iron. Notably, iron in PO, present at concentrations of 0.13–0.15 mg/g, is a vital trace element. Iron plays a key role in Hb synthesis, and its deficiency can reduce Hb levels. In fish, iron deficiency impairs Hb synthesis and erythropoiesis, resulting in decreased RBC production, microcytosis (smaller RBCs), and lower Hct levels. Therefore, it is plausible that the dietary supplementation of PO enhanced the health condition of the Nile tilapia in this study. WBCs are the primary component of the nonspecific immune defense system in fish, which act as the first line of defense against various threats and pathogens [82]. In the present study, WBC levels exhibited a quadratic increase as the PO level increased, with the highest level observed in the PO50 group. These findings align with the previous study that demonstrated the supplementation of water hyacinth leaf powder (*Eichhornia crassipes*) containing high levels of phenolic compounds and flavonoids in pikeperch (*Sander lucioperca*) led to a notable increase in RBCs, WBCs, and Hb levels [83]. Similarly, fish fed *Annona squamosa* leaves [84], *Moringa oleifera* leaf [85], and *Urtica dioica* leaf [86], significantly increased these hematological values. Nevertheless, the effect of PO and hematology needs further investigation.

In the present study, the levels of total protein, albumin, and globulin increased at higher levels of PO supplementation. The highest levels were observed in the PO50 group with the lowest A:G ratio. Generally, a high level of total protein in the blood signifies good health in fish since serum levels of albumin and globulin, along with a low A:G ratio, serve as indicators of strong innate immunity [87–89]. ALP, ALT, and AST are also common parameters of fish health that are sensitive to liver function and damage [90–92]. In the present study, dietary supplementation of PO significantly decreased ALT activity but did not significantly affect AST and ALP activities [32]. There is limited information on the protective effects of PO supplementation on hepatic enzymes in fish. However, some studies reported that serum levels of AST and ALT were lower in the fish fed herbal immunostimulants such as mistletoe, mushroom, and *Aegle marmelos* [93, 94]. Overall, these findings suggest that hepatic injury or damage caused by PO supplementation is limited at least under tested conditions. Serum triglyceride and LDL-c levels exhibited a quadratic decrease with increasing PO supplementation levels, whereas an opposite effect was observed for HDL-c levels. The alterations observed in serum triglyceride, LDL, and HDL levels imply that the phytochemicals in PO influence the lipid metabolism of Nile tilapia, potentially promoting fish health.

Inflammation is a key part of the body's defense against infections and other causes, classified into acute, chronic, and subacute types based on response duration [95]. It also engages both innate and adaptive immune components in fish [96]. ILs are signaling molecules that regulate immune responses in infectious diseases, playing roles in immune cell recruitment and activation, production of proinflammatory cytokines, and modulation of both adaptive and innate immunity in fish. Proinflammatory ILs activate and recruit immune cells, while anti-inflammatory ILs suppress excessive inflammation and promote tissue repair [94]. IL-1β and TNF-α are the initial proinflammatory cytokines released in response to pathogens, which play a crucial role in initiating the acute phase of the innate immune response. These cytokines achieve the effect by enhancing vascular permeability and by facilitating the recruitment of inflammatory cells [97] and also stimulate the expression of several genes associated with inflammation such as IL-1β, IL-8, IL-17C, TNF-α, and COX-2 [97–99]. In addition, IL-1β and TNF-α influence genes involved in antimicrobial responses, enhance phagocytic and leucocyte activities, and boost the production of reactive oxidant species used for the defense against bacteria [99, 100]. IL-10 and TGF-β are anti-inflammatory and regulatory cytokines with versatile roles in the immune system. They not only inhibit the excessive release of cytokines but also control various inflammatory processes, highlighting their essential roles in immune regulation [101, 102]. Our study showed that dietary supplementation of PO upregulated IL-1β, IL-10, TNF-α, and TGF-β mRNA expression with the highest levels of IL-1, IL-10, and TNF-α observed in the PO30 group. The PO is rich in phytochemical constituents such as total phenolic compounds (58.56 μg/mg), flavonoid contents (70.65 μg/mg), and over 23 volatile essential oil compounds [103–105]. These compounds are believed to promote anti-inflammatory, antioxidant, antimicrobial, and antitumor properties in fish [106, 107]. A previous study also showed that methanol extract of crude PO could activate the defense against *Staphylococcus aureus* isolated from fish [108]. In addition, dietary supplementation of thyme and basil oil, containing high levels of phenolic, flavonoid, and essential oil, enhanced proinflammatory cytokine genes such as TNF-α, IL-1β, IL-6, TGF-β, and IL-8 in rainbow trout [109, 110]. The upregulation of pro- and anti-inflammatory cytokine genes may indicate the enhanced immune response and fish's ability to resist pathogens.

HSP70 enhances the immune response and actively participates in safeguarding cytoplasmic components against stress under diverse stressful conditions [48, 111]. The increase in HSP70 levels contributes to modulating cellular antistress responses and especially plays a key role in protecting organisms against heat stress [112, 113]. In the present study, the expression of HSP70 in the fish fed dietary supplementation of PO was significantly higher than that of the control group (PO0). This gene upregulation may suggest an enhancement in the immunity of Nile tilapia. Similar observations have been reported for many herbal supplementations such as hesperidin extract of citrus fruit, thyme powder, Codonopsis *pilosula,* and a combination of guava, bitter, and neem leaf extracts [114–117]. However, further research is necessary to explore the effects of PO on HSP70 expression in fish, as current studies are limited.

Glucose and cortisol are practical indicators of both primary and secondary stress responses, which rise in response to stress and decrease during phases of improved well-being [118–120]. The present study showed that dietary supplementation of PO50 significantly reduced blood glucose levels, and all PO supplementation groups exhibited significantly lower levels of cortisol compared to the control group. The decrease in blood glucose and cortisol levels could be attributed either to the stimulation of hypoglycemic hormones such as insulin or to a reduction in glucose absorption [121, 122]. The lowered level of glucose and reduced cortisol levels observed in the PO-supplemented groups of the present study suggest that dietary supplementation of POs has an antistress effect in Nile tilapia. This effect is likely attributed to its active compounds pinenes, known for their sedative, antidepressant, and analgesic properties [123, 124]. However, this requires further studies because there have been no specific studies conducted to assess the impact of PO supplementation on these properties in fish. Similar effects antistress effects have been observed in Nile tilapia for pinenes in lemon peels [125] as well as for other herbal feed additives such as turmeric extract [50], garlic [39], bitter lemon peel extract [40, 125], mooseer (*Allium hirtifolium*) [126], and red pepper [41].

## 5. Conclusion

In summary, dietary PO supplementation enhanced growth performance, feed utilization, and blood hematological and biochemical parameters, mitigating stress markers in Nile tilapia. These effects would be attributed at least partly to the enhanced expression of immune-related proinflammatory and anti-inflammatory genes. The recommended dosage of PO supplementation is 30 g/kg for the higher growth performance (FBW, WG, and SGR), feed efficiency (PER and PPV), blood biochemistry (higher HDL-c), and expression of gene related to immunity (IL-1β, IL-10, and TNF-α). These results provide compelling evidence that PO holds immense promise as an immunostimulant, which effectively enhances the immune capabilities of Nile tilapia and boosts the overall production in tilapia farming. It also supports eco-friendly disease management, promoting sustainability in intensive farming systems.

## Figures and Tables

**Table 1:** Formulation and proximate composition of the experimental diets (g/kg, dry matter).

Ingredients	PO0	PO15	PO30	PO50
Feed formula (g·kg^−1^)^a^				
Fish meal^b^	135	135	135	135
Soybean meal^c^	400	400	400	400
Squid meal^d^	33	33	33	33
Rice bran	187	187	187	187
Wheat flour	70	55	40	20
Corn meal	50	50	50	50
Broken rice meal	60	60	60	60
Vitamin premix^e^	10	10	10	10
Mineral premix^f^	10	10	10	10
Soybean oil	22	22	22	22
*Persicaria odorata*	0	15	30	50
Lecithin	4	4	4	4
Vitamin C	5	5	5	5
Methionine	9	9	9	9
Lysine	5	5	5	5
Total	1000	1000	1000	1000

Composition (%)^g^				
Crude protein	31.71	31.75	30.37	31.66
Crude fat	7.06	7.33	7.03	7.10
Ash	4.36	4.42	3.90	3.84
Crude fiber	4.04	4.21	5.59	5.62
Moisture	6.98	7.05	7.50	7.25

^a^The experimental diets were formulated to be isonitrogenous (∼31%) and isolipidic (∼7%) for all four diets.

^b^Fish meal, Phraepan Animal Feed Distributor, Phitsanulok, Thailand.

^c^Soy protein concentrate, Thai Vegetable Oil PCL Co, Ltd., Nakorn Pathom, Thailand.

^d^Squid meal, Pro-Squid Co, Ltd., Bangkok, Thailand.

^e^Vitamin mixture, SUN-MIX, Muankkong Interfood, Co, Ltd., Nakorn Pathom, Thailand.

^f^Mineral mixture, MINMIN, Muankkong Interfood, Co, Ltd., Nakorn Pathom, Thailand.

^g^Crude protein and crude fat contents were formulated to meet the optimal nutritional requirements of Nile tilapia [39, 40].

**Table 2:** Primer sequences used in the present study.

Genes	Sequences (5′–3′)	References
IL-1β	F-AAGATGAATTGTGGAGCTGTGTT	[47]
	R-AAAAGCATCGACAGTATGTGAAAT	

IL-10	F-TGGAGGGCTTCCCCGTCAG	[48]
	R-CTGTCGGCAGAACCGTGTCC	

TNF-α	F-GCTGGAGGCCAATAAAATCA	[49]
	R-CCTTCGTCAGTCTCCAGCTC	

TGF-β	F-TGCGGCACCCAATCACACAAC	NM_001311325.1
	R-GTTAGCATAGTAACCCGTTGGC	

HSP70cB1	F-CTCCACCCGAATCCCCAAAA	[50]
	R-TCGATACCCAGGGACAGAGG	

β-Actin	F-TGGTGGGTATGGGTCAGAAAG	XM_003443127
	R-TGTTGGCTTTGGGGTTCA	

**Table 3:** Growth performance, feed utilization, survival, and organosomatic indexes of tilapia fed graded levels of *Persicaria odorata* for 10 weeks.

Parameters	PO0	PO15	PO30	PO50	SEM	Linear	Quadratic
Growth and feed utilization							
IBW (g/fish)	7.78 ± 0.02	7.77 ± 0.01	7.78 ± 0.01	7.77 ± 0.01	< 0.001	0.275	1.000
FBW (g/fish)	76.28 ± 3.30	81.12 ± 1.34⁣^∗^	84.16 ± 1.77⁣^∗^	81.64 ± 2.09⁣^∗^	5.045	0.011	0.022
WG (g/fish)	68.50 ± 3.30	73.35 ± 1.34⁣^∗^	76.39 ± 1.77⁣^∗^	73.87 ± 2.10⁣^∗^	5.060	0.011	0.022
SGR (%/day)	3.26 ± 0.06	3.35 ± 0.02⁣^∗^	3.40 ± 0.03⁣^∗^	3.36 ± 0.04⁣^∗^	0.001	0.011	0.043
FCR	0.95 ± 0.03	0.93 ± 0.02	0.95 ± 0.02	1.02 ± 0.04⁣^∗^	0.002	0.009	0.028
PER	1.87 ± 0.01	1.99 ± 0.01⁣^∗^	2.14 ± 0.02⁣^∗^	1.96 ± 0.01⁣^∗^	0.025	< 0.001	< 0.001
PPV (%)	23.94 ± 0.21	26.96 ± 0.09⁣^∗^	27.63 ± 0.32⁣^∗^	25.76 ± 0.12⁣^∗^	0.229	< 0.001	< 0.001
Survival (%)	98.33 ± 2.89	98.33 ± 2.89	100.00 ± 0.00	100.00 ± 0.00	< 0.001	0.592	0.823
Organosomatic indexes							
K (g/cm^3^)	1.93 ± 0.14	1.96 ± 0.22	1.97 ± 0.13	1.82 ± 0.14	0.027	0.203	0.109
HSI (%)	1.57 ± 0.44	1.63 ± 0.51	1.68 ± 0.46	1.75 ± 0.53	0.236	0.428	0.953
VSI (%)	7.55 ± 0.89	7.85 ± 1.65	8.18 ± 0.72	8.39 ± 0.99	1.249	0.097	0.902

*Note:* Growth and feed utilization are mean ± SD (*n* = 60; 20 fish from each tank). Organosomatic indexes are mean ± SD from three replicate treatments (*n* = 9; three fish from each tank). K, condition factor.

Abbreviations: FBW, final body weight; FCR, feed conversion ratio; HSI, hepatosomatic index; IBW, initial body weight; PER, protein efficiency ratio; SGR, specific growth rate; VSI, viscerosomatic index; WG, weight gain.

⁣^∗^Significant differences from the control group (PO0, Dunnett's test, *p* < 0.05).

**Table 4:** Whole-body composition of Nile tilapia fed different levels of *Persicaria odorata* for 10 weeks.

Composition (%)	PO0	PO15	PO30	PO50	SEM	Linear	Quadratic
Moisture	77.43 ± 0.51	77.17 ± 1.55	78.22 ± 0.80	77.37 ± 0.35	0.615	0.885	0.827
Crude protein	53.21 ± 0.60	55.10 ± 0.26⁣^∗^	55.17 ± 0.80⁣^∗^	54.14 ± 1.41	0.623	0.222	0.017
Crude fat	30.77 ± 0.16	31.24 ± 0.63	33.31 ± 0.08⁣^∗^	31.48 ± 0.82	0.184	< 0.001	< 0.001
Ash	12.68 ± 0.14	13.47 ± 0.44	13.38 ± 0.96	13.19 ± 0.19	0.552	0.267	0.084

*Note:* Data are represented as mean ± SD values (*n* = 3 per treatment; 3 fish from a tank were pooled).

⁣^∗^Significant differences from the control group (PO0, Dunnett's test, *p* < 0.05).

**Table 5:** Hematological characteristics of Nile tilapia fed different levels of *Persicaria odorata* meal for 10 weeks.

Hematological parameters	PO0	PO15	PO30	PO50	SEM	Linear	Quadratic
Red blood cell (× 10^6^ cell/μL)	2.01 ± 0.52	2.05 ± 0.27	2.47 ± 0.12⁣^∗^	2.76 ± 0.18⁣^∗^	0.096	0.576	0.007
White blood cell (× 10^3^ cell/μL)	2.75 ± 0.24	3.97 ± 0.76⁣^∗^	3.10 ± 0.28	4.26 ± 0.47⁣^∗^	1.084	0.069	0.038
Hematocrit (%)	24.78 ± 1.55	26.30 ± 2.89	29.99 ± 1.92	28.82 ± 1.26	4.561	0.073	0.091
Hemoglobin (g/dL)	10.85 ± 1.14	11.75 ± 0.90	12.32 ± 0.38⁣^∗^	12.24 ± 0.28⁣^∗^	0.900	0.510	0.004

*Note:* Hematological parameters are mean ± SD values (*n* = 3; 3 fish from each tank were pooled).

⁣^∗^Significant differences from the control group (PO0, Dunnett's test, *p* < 0.05).

**Table 6:** Blood biochemical characteristics of Nile tilapia fed different levels of *Persicaria odorata* meal for 10 weeks.

Items	PO0	PO15	PO30	PO50	SEM	Linear	Quadratic
Total protein (g/dL)	3.42 ± 0.04	3.42 ± 0.13	3.58 ± 0.08	3.88 ± 0.03⁣^∗^	0.008	0.001	0.035
Albumin (g/dL)	1.11 ± 0.02	1.12 ± 0.12	1.14 ± 0.05	1.19 ± 0.01	0.006	0.311	0.345
Globulin (g/dL)	2.31 ± 0.02	2.37 ± 0.05	2.49 ± 0.06⁣^∗^	2.69 ± 0.02⁣^∗^	0.004	< 0.001	0.005
A:G ratio	0.48 ± 0.01	0.47 ± 0.05	0.46 ± 0.02	0.44 ± 0.00	0.002	0.135	0.727
AST (U/L)	37.18 ± 4.78	27.91 ± 4.56	31.82 ± 4.65	29.94 ± 2.84	8.316	0.125	1.74
ALT (U/L)	33.39 ± 3.08	22.16 ± 4.01⁣^∗^	24.47 ± 5.07	29.18 ± 3.02	5.072	0.018	0.007
ALP(U/L)	16.29 ± 6.51	22.40 ± 4.13	28.95 ± 6.55	21.42 ± 2.26	6.846	0.140	0.052
Total cholesterol (mg/dL)	144.85 ± 10.20	132.19 ± 3.02	141.62 ± 4.78	131.83 ± 3.55	7.122	0.096	0.695
Triglyceride (mg/dL)	102.61 ± 9.51	72.96 ± 6.17⁣^∗^	80.18 ± 6.99⁣^∗^	99.16 ± 8.71	3.000	0.882	0.020
HDL-c (mg/dL)	68.74 ± 5.04	75.20 ± 2.98	89.35 ± 2.04⁣^∗^	77.69 ± 3.43⁣^∗^	12.551	0.002	0.002
LDL-c (mg/dL)	52.39 ± 2.61	44.82 ± 1.60⁣^∗^	45.53 ± 3.38⁣^∗^	49.35 ± 0.74	5.333	0.193	0.003

*Note:* Data are mean ± SD values (three fish from each tank were pooled, *n* = 3).

⁣^∗^Significant differences compared to the control group (PO0, Dunnett's test, *p* < 0.05).

**Table 7:** Blood glucose and cortisol levels of Nile tilapia fed different levels of *Persicaria odorata* meal for 10 weeks.

Items	PO0	PO15	PO30	PO50	SEM	Linear	Quadratic
Hematological parameters							
Glucose (mmol/L)	78.67 ± 4.04	79.33 ± 3.51	73.33 ± 1.15	67.00 ± 2.00⁣^∗^	2.375	0.010	0.071
Cortisol (mg/mL)	2.52 ± 0.20	1.86 ± 0.15⁣^∗^	1.76 ± 0.18⁣^∗^	1.77 ± 0.08⁣^∗^	0.166	< 0.001	0.016

*Note:* Data are mean ± SD values (three fish from each tank were pooled, *n* = 3).

⁣^∗^Significant differences when compared to the control group (PO0, Dunnett's test, *p* < 0.05).

## Data Availability

The datasets utilized and examined in the present study can be obtained from the corresponding author upon reasonable request.
